# Drug-protein interaction prediction *via* variational autoencoders and attention mechanisms

**DOI:** 10.3389/fgene.2022.1032779

**Published:** 2022-10-14

**Authors:** Yue Zhang, Yuqing Hu, Huihui Li, Xiaoyong Liu

**Affiliations:** School of Computer Science, Guangdong Polytechnic Normal University, Guangzhou, China

**Keywords:** drug-protein interactions (DPIs), variational autoencoder (VAE), attention mechanism, convolutional neural network (CNN), deep learning - artificial neural network

## Abstract

During the process of drug discovery, exploring drug-protein interactions (DPIs) is a key step. With the rapid development of biological data, computer-aided methods are much faster than biological experiments. Deep learning methods have become popular and are mainly used to extract the characteristics of drugs and proteins for further DPIs prediction. Since the prediction of DPIs through machine learning cannot fully extract effective features, in our work, we propose a deep learning framework that uses variational autoencoders and attention mechanisms; it utilizes convolutional neural networks (CNNs) to obtain local features and attention mechanisms to obtain important information about drugs and proteins, which is very important for predicting DPIs. Compared with some machine learning methods on the C.elegans and human datasets, our approach provides a better effect. On the BindingDB dataset, its accuracy (ACC) and area under the curve (AUC) reach 0.862 and 0.913, respectively. To verify the robustness of the model, multiclass classification tasks are performed on Davis and KIBA datasets, and the ACC values reach 0.850 and 0.841, respectively, thus further demonstrating the effectiveness of the model.

## Introduction

Finding gene-drug relationships is important not only for understanding a certain mechanism of drug molecules, but also for developing treatments for patients. The gene-drug relationship is many-to-many, which is much more complex than a gene-to-drug or a drug-to-gene, and also explains the complex relationship between gene-drug. The gene-drug relationship has similarities to the drug-protein relationship (Chen et al., 2019; Huang et al., 2021).

In the prediction of RNA-binding proteins, limited by the huge cost of biological experiments, it is difficult to fully understand the underlying mechanisms of alternative splicing (AS) and related RNA-binding proteins (RBPS) in regulating the epithelial-mesenchymal transition (EMT) process. This needs to be achieved by means of computational methods (Qiu et al., 2021b) proposed an inductive matrix-based model to study the relationship between RBP and AS during EMT. The main purpose of the model is to compensate for missing and unknown RBP-AS relationships (Qiu et al., 2021a) proposed a method based on weighted data fusion with sparse matrix tri-factorization to conduct experiments. The AS-RBP relationship is explored by assigning different weights to the source data. Both methods achieve good results. At the same time, this has parallels with the drug-protein relationship. It achieves the desired effect by looking for a drug to inhibit an binding site of a protein.

Drug-protein interactions (DPIs) exploration is a critical step in the drug discovery process. With the discovery of new drugs, the field of drug development continues to expand, and awareness regarding the repositioning of existing drugs and new interactions involving approved drugs is of increasing concern (Oprea and Mestres, 2012). Based on biological experiments, it usually takes 10–20 years and much money (US$ 0.5–260 million) to develop a new drug (Avorn, 2015), so it is important to explore the interactions between drugs and proteins. In recent years, computer-aided methods have achieved good results and contributed significantly to the prediction of DPIs. The application of artificial intelligence in chemical research can accelerate the development of high-precision DPIs prediction methods.

In the past decade, the problem of predicting the interactions between drugs and proteins has been solved using traditional machine learning methods, which solve binary classification problems (Yamanishi et al., 2010; Liu et al., 2016; Nascimento et al., 2016; Keum and Nam, 2017). Due to the rise and popularity of deep learning, it has become a popular choice for solving DPIs predictions (Unterthiner and Mayr, 2014; Tian et al., 2016) used a deep neural network (DNN) to explore the interactions between drugs and proteins instead of traditional machine learning methods, which directed the subsequent research on drug and protein interactions toward deep learning approaches, such as convolutional neural networks (CNNs), recurrent neural networks (RNNs) (Gao et al., 2018; Mayr et al., 2018) and stacked autoencoders (Wang et al., 2018).

In general, DPIs approaches can be divided into three categories: docking-based methods, machine learning-based methods, and deep learning-based methods. Docking-based methods require the best site and protein structure to be found and combined, but such a technique usually time-consuming, and many datasets lack three-dimensional protein structures (Gschwend et al., 1996). Machine learning-based methods (Faulon et al., 2008; Bleakley and Yamanishi, 2009; Ballester and Mitchell, 2010) usually require manual features, and the features passed to the model before modeling occurs require manual participation, which demands considerable feature extraction experience and expertise. Deep learning-based methods have been applied to many fields in biology (Min et al., 2016; Zeng et al., 2019; Zhang et al., 2019, 2021; Wu et al., 2022b); DPIs prediction performance has been improved through the framework structure and network parameters of deep learning. For example, the DeepDTA approach of (Öztürk et al., 2018) learns internal high-level features by extracting the features of drugs and proteins as the network inputs and then predicts the relationships between drugs and proteins. The WideDTA method proposed by (Öztürk et al., 2019) is similar to DeepDTA, and the network framework is roughly unchanged; the main difference is that when inputting features, WideDTA extracts the features of drug proteins from multiple aspects as model inputs. Notably, a graph-based network architecture called GraphDTA (Nguyen et al., 2019), which treats drugs as a graph structure to predict DPIs, has also been developed. A Novel Graph Neural Network for Predicting Drug-Protein Interactions called BridgeDTA (Wu et al., 2022a), which introduces a class of nodes named hyper-nodes, which bridge different proteins/drugs to work as the protein-protein and drug-drug associations. HOGMMNC is a higher order graph matching with multiple network constraints model. It mainly obtains the fixed structural relationship in multi-source data through hypergraph matching, so as to identify the relationship between genes and drugs, and improve the accuracy and reliability of the identification relationship (Chen et al., 2019). These deep learning methods all have three similarities. *1*) They encode drugs and proteins. *2*) They extract the high-level features of drugs and proteins through their network structures. *3*) They predict the features obtained in *2*) through a fully connected (FC) layer. The advantage of these methods is that the process is not too cumbersome (it is simple). Furthermore, we exploit the strengths of these network frameworks for the prediction of DPIs.

A variational autoencoder (VAE) is a machine learning model that can reconstruct a variable x based on a latent feature Z . Unlike a simple autoencoder, it can learn the distribution of latent variables and then sample from this distribution to generate new samples. The model has been shaped and used in various fields, such as image processing (Walker et al., 2017; Liu et al., 2018) and text processing (Mayr et al., 2018). We use this model to predict DPIs. Experimental results show that better results are achieved on some datasets. Our contributions are as follows.

1) A variational autoencoder is designed to provide a probabilistic way of describing the latent representation of drugs and proteins, denoted *via* mean and variance of the hidden state distribution. Such generative way effectively reduce the redundant information in the raw samples to ease for leaning drug-protein interactions.
2) Discriminative local features on drugs and proteins are extracted *via* deep CNN. A specially designed attention mechanism is incorporated to focus on the key interactive information on both drugs and proteins, thus obtaining strong drug-to-drug and protein-to-protein relationships.
3) Extensive experiments on C.elegans and Human dataset, BindingDB dataset, Davis dataset and KIBA dataset. Datasets demonstrate that the proposed method can robustly identify the drug-protein interactions.

## Methods

### A VAE network to identify drug and protein interactions

**FIGURE 1:**
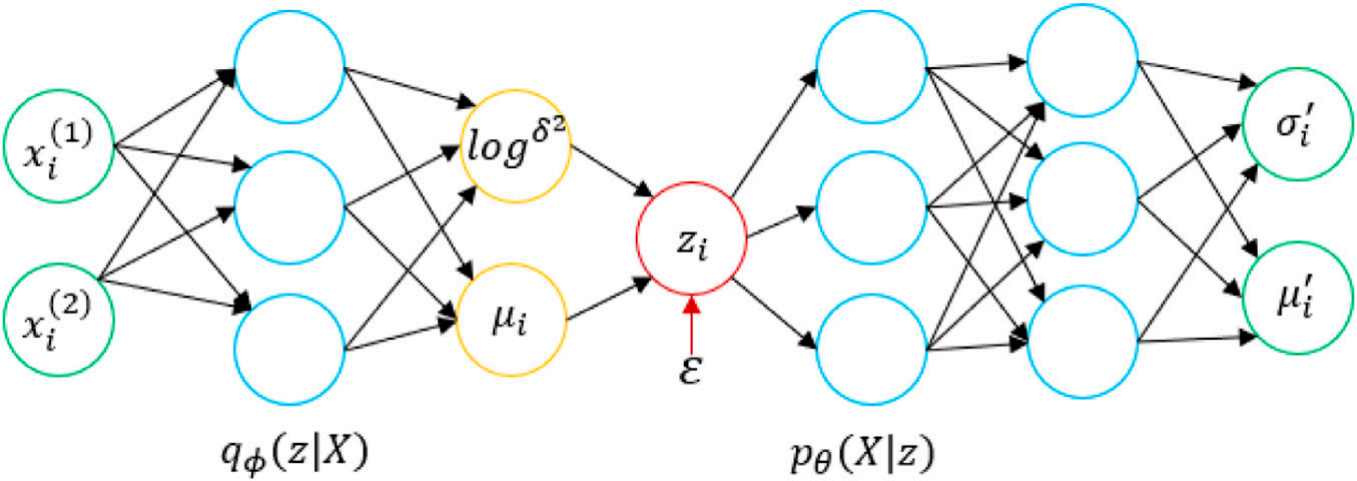
Structural diagram of the utilized VAE.

We input a set of drug molecules D and a protein sequence T, and a VAE (Kingma and Welling, 2014) learns the distribution of a multidimensional variable x based on an independent and identically distributed latent variable $(X={\left\{x^{i}\right\}}_{1}^{N}$ , where *N* is the number of samples). The framework of this strategy is shown in Figure 1, where $x_{i}^{\left(j\right)}$ represents the jth feature of the *ith* sample.

First, a data point $x_{i}$ is input into the encoder. Through the neural network, we obtain the parameters of the approximate posterior distribution $q_{\emptyset }\left(z|x_{i}\right)$ obeyed by the hidden variable z . The posterior distribution is a Gaussian distribution, and the output of the encoder includes the parameters $\sigma_{i}^{2}$ and $\mu_{i}$ of the Gaussian distribution that $z|x_{i}$ obeys. With the parameters $\sigma_{i}^{2}$ and $\mu_{i}$ of the $z|x_{i}$ distribution, we sample one $\epsilon_{i}$ from N(0,I) and use the reparameterization trick to set $z_{i}=\mu_{i}+\sigma_{i}⨀\epsilon_{i}$ where $z_{i}$ represents the value of a similar sample $x_{i}$ and ⨀ represents the elementwise multiplication operation. The decoder needs to fit the likelihood distribution $p_{\emptyset }\left(X|z_{i}\right)$ and feed a $z_{i}$ to the decoder, which returns the parameters of the distribution that $X|z_{i}$ obeys; the likelihood also obeys a Gaussian distribution. After obtaining the parameters of the distribution of $|z_{i}$ , we sample from the distribution to generate a sample $x_{i}$ . In the last step, we do not sample and directly regard the $\mu_{i}^{\prime }$ output by the model as the sample $x_{i}$ generated by $z_{i}$ . Then, we can obtain the objective function of the VAE, and we only need to maximize L .

$$L\left(p_{\theta },q_{\emptyset }\right)=-D_{KL}\left(q_{\emptyset },p\right)+E_{q_{\emptyset }}\left[\log p_{\theta }\left(X|z\right)\right]$$

$-D_{KL}\left(q_{\emptyset },p\right)$ is the Kullback-Leibler (KL) divergence of the two distributions p and q and is also a regular term. $E_{q_{\emptyset }}\left[\log p_{\theta }\left(X|z\right)\right]$ is often referred to as the reconstruction loss.

With the theoretical support of the VAE, we apply it to the prediction of DPIs. Here, $x_{d}$ and $x_{t}$ represent the drug and protein vectors, respectively, and y represents the interaction relationship or affinity value between drug and protein. We assume that the hidden variables of $x_{d}$ and $x_{t}$ are $z_{d}$ and $z_{t}$ , respectively. Our aim is to learn a model that predicts drug and protein interactions.

**FIGURE 2:**
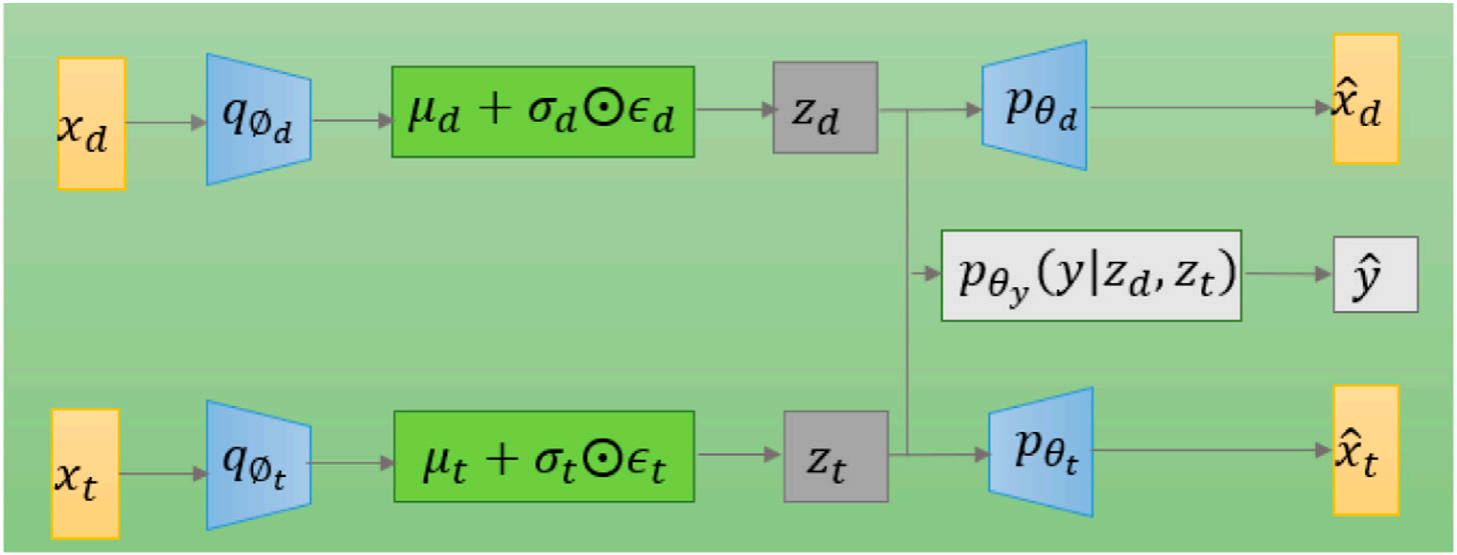
The graphical structure of the VAE model for DPIs prediction.

The model diagram for applying variational autoencoding to DPIs prediction is shown in Figure 2. The model has two encoders, which are mainly used to generate latent variables $z_{d}$ and $z_{t}$ for drug $x_{d}$ and protein $x_{t}$ , respectively. Three important decoders are used to generate $x_{d}$ and $x_{t}$ from the latent variables $z_{d}$ and $z_{t}$ and generate the drug-protein relationship y . Finally, for each drug-protein pair, the objective function of the VAE model is to maximize $L^{\prime }$ .

$$L^{\prime }=L_{DrugVAE}+L_{PorteinVAE}$$

$$=\left\{-D_{KL}\left(q_{\emptyset_{d}},p_{d}\right)+E_{q_{\emptyset_{d}}}\left[\log p_{\theta_{d}}\left(X_{d}|z_{d}\right)\right]\right\}$$

$$+\left\{-D_{KL}\left(q_{\emptyset_{t}},p_{t}\right)+E_{q_{\emptyset_{t}}}\left[\log p_{\theta_{t}}\left(X_{t}|z_{t}\right)\right]\right\}$$

### Attention mechanism for feature extraction

Attention mechanisms, as effective means of feature screening and enhancement, have been widely used in many fields of deep learning. A structural model based on an attention mechanism can not only record the positional relationships between pieces of information but also measure the importance levels of different information features according to the weight of the information. Dynamic weight parameters are established by making relevant and irrelevant choices for the information features to strengthen the key information and weaken the useless information, thereby improving the efficiency of deep learning algorithms and improving some of the defects of traditional deep learning techniques.

Utilizing an attention mechanism for the prediction of DPIs can enable effective atomic feature extraction because the structures of the molecular sequences of drugs and proteins are very similar to the structures of natural language sentences, and the context information of atoms is very important for understanding molecular features (Jastrzębski et al., 2018). In detail, we should pay attention to the interaction information of each atom and its adjacent atoms; each atom is also connected to the simplified molecular-input line-entry system (SMILES (Weininger, 1988), which is a symbol for molecular structure encoding). Information about the interactions of atoms that are farther away in the sequence can also have an impact on the predicted results. The molecular sequences of proteins are very long, and the best way to extract features is to use an attention mechanism.

Attention mechanisms are widely used in the natural language processing (NLP) field, and they have also been shown to be powerful for processing textual data. The core of such a mechanism is an attention function (Vaswani et al., 2017). The attention function can be described as mapping a query (Q) and a set of key-value (K-V) pairs to an output. Among them, dot product attention with Q, K and V is a widely used attention approach. Let the dimensions of Q and K be $d_{k}$ and the dimensionality of V be $d_{v}$ . Then, attention can be expressed by the following formula.

$$Attenton\left(Q,K,V\right)=softmax\left(\frac{QK^{T}}{\sqrt{d_{k}}}\right)V$$

where $Q\in R^{n\times d_{k}},K\in R^{m\times d_{k}}$ and $V\in R^{m\times d_{v}}$ . The attention measures the similarity between the inner product of the matrix Q and V , which is nonlinearly transformed by a softmax function with the matrix V . Here, $d_{k}$ prevents the forward propagation of the network due to excessive data content.

### The network structure of the model

**FIGURE 3:**
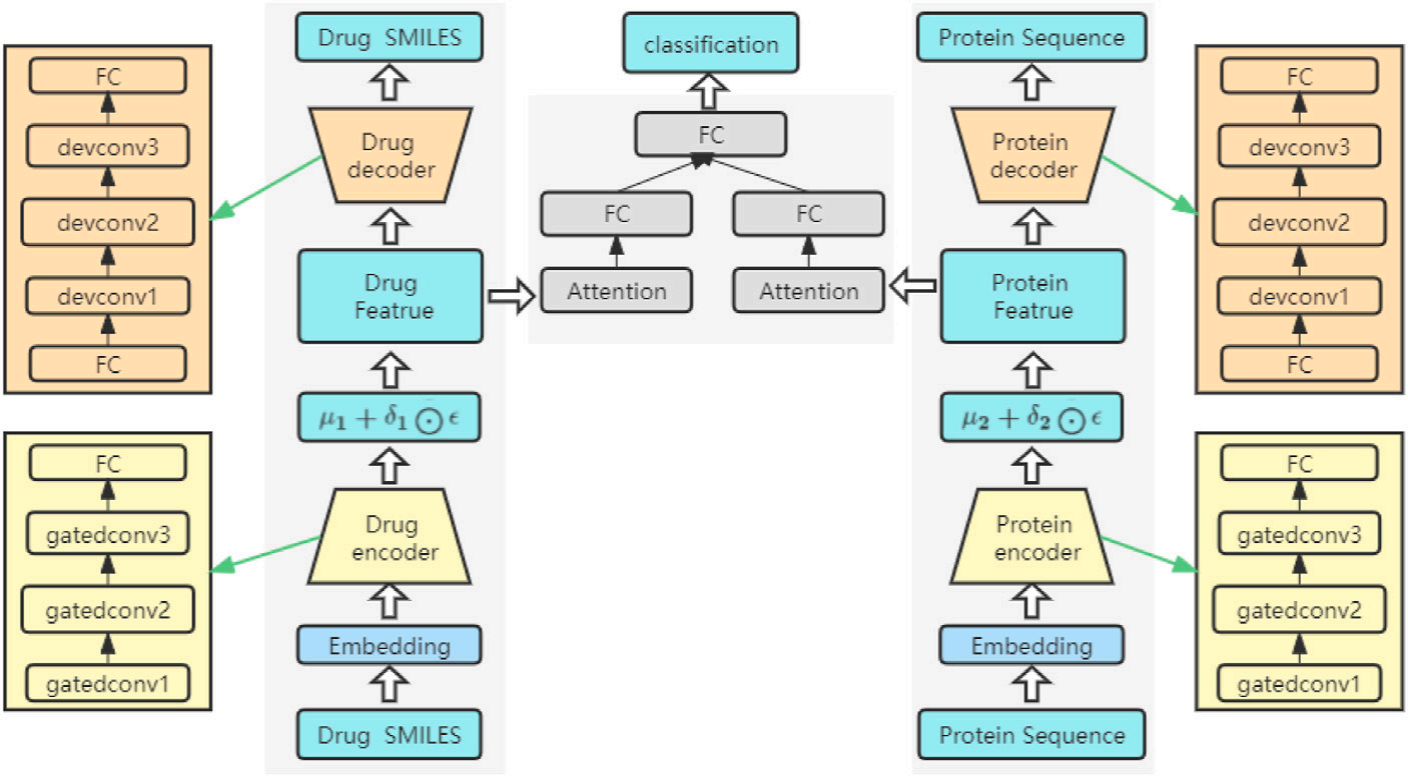
The framework structure of the model.

The network structure of the model is shown in Figure 3. It consists of three key parts: an encoder, a decoder and a prediction module. Both the encoder and the decoder serve to predict the interactions between drugs and proteins. Among them, the overall structure extracts the features of drugs and proteins, sends the extracted features into the attention block to focus on the important parts, and finally sends them to the FC layer to predict the DPIs. The feature extraction process for drugs is the same as that for proteins. Before being fed into the encoder, drugs and proteins are sequences of text strings, which need to be converted into digital vectors. According to the existing character dictionary, each character is converted into an integer type, and then each sample is converted into an embedding matrix through embedding. In our model, three GatedCNNs are included in the coding layer, and a rectified linear unit (RELU) (Nair and Hinton, 2010) activation function is present after each layer of CNNs. The filters of the last two CNNs are the first CNNs, which are filtered two times and three times. A max pooling layer is appended after the third GatedCNN to compress the extracted features.

In the decoders for drugs and proteins, the input source data are reconstructed through deconvolutional networks (Zeiler et al., 2010). Each decoder has an FC layer and three deconvolution layers. The last deconvolution appends an FC layer to convert the output into drug and target sequences with the same size as that of the input.

In the DPIs prediction module, two FC layers are used to represent the features of drugs and proteins. To further extract the high-level features of drugs and proteins, a self-attention mechanism is introduced after the FC layers, focusing on important features in drug sequences or protein sequences and ignoring unnecessary features. Then, the final extracted high-level features are spliced and sent to a network containing three FC layers. A ReLU activation function and a dropout function are placed after the first two FC layers, and the dropout function is mainly used to prevent the network from overfitting. The final output can be used to predict DPIs.

The model parameters used in this experiment are shown in Table 1. Among them, we select several values [16,32,64] for the number of CNN filters in the encoder and decoder and find that the effect of 32 filters was best and that the filter lengths of drugs and proteins are both in [5,7,9,11]. We choose the best results, and the final filter lengths of drugs and proteins are 5 and 7, respectively.

**TABLE 1:** Model parameters.

Parameter	Value
Number of filters in the encoder and decoder	32
Filter length (drug molecules)	5
Filter length (protein sequences)	7
Number of epochs	300
Batch size	256
Learning rate	0.001, 0.0001

## Experiment

### Datasets

To verify the effectiveness of the proposed model and compare it with the base method, we conducted experiments on the following datasets: C.elegans and Human datasets, BindingDB dataset, Davis dataset and KIBA dataset.

#### C.elegans and human datasets

In the work of (Liu et al., 2015), the authors used a systematic scanning framework, and their dataset contained a large number of negative samples. They constructed two datasets, C.elegans and human. Following the requirements of (Tsubaki et al., 2019), we used a balanced dataset with an approximately 1:1 ratio of positive and negative samples. The C.elegans dataset includes 1876 protein targets and 1767 drug molecules, and it contains 7786 affinity sample pairs, 3893 positive samples, and 3893 negative samples. The human dataset contains 6728 affinity pairs, the number of protein targets is 2001, and the number of drug molecules is 2726.

#### BindingDB dataset

BindingDB is a public, web-accessible database of measured binding affinities that focuses chiefly on the interactions of proteins considered to be drug targets with small, drug-like molecules. In this experiment, the method described in the paper of (Gao et al., 2018) was used; the dataset contains 39,747 positive samples and 31,218 negative samples.

#### Davis

**FIGURE 4:**
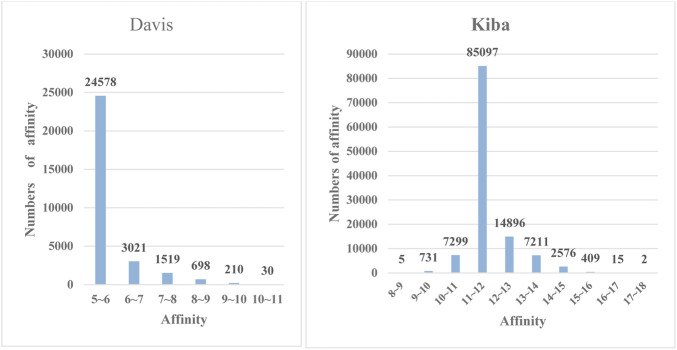
The frequency histograms of the affinities in the Davis and KIBA datasets. The horizontal axis denotes the affinity values of drugs and proteins, and the vertical axis represents the numbers of affinity values in certain intervals.

The Davis dataset contains affinity pairs measured by their $K_{d}$ value (kinase dissociation constant); it includes 68 drug molecules and 442 proteins (Davis et al., 2011). The $K_{d}$ value can reflect the affinity between a drug and a protein. It is a bridge for predicting affinity, and its affinity value range is [0.016, 10000]. The frequency distribution plot of affinity values for the Davis dataset is shown in Figure 4.

#### KIBA

Measuring the relationships between the drugs and proteins in the KIBA dataset is mainly achieved through the KIBA score (Tang et al., 2014) combined the biological activities of different sources of kinase inhibitors, such as $K_{d}$ , $K_{i}$ and IC50 , to calculate KIBA affinity scores. We conducted experiments according to this environment. Then, we found that the dataset contains 52498 drug molecules, 476 protein sequences, and 246088 KIBA scores and that the numerical range of the KIBA score is [0, 17.2]. Values in this dataset with KIBA scores that were less than 10 were removed, leaving 2111 drug molecules and 229 proteins in the end. The frequency distribution plot of the affinity values for the KIBA dataset is shown in Figure 4.

### Training details

The model was implemented based on Python 3.6 and PyTorch 1.10.2. The program ran on a GTX1060 GPU with 8 GB of memory. The network parameter initialization process was implemented by the *xavier_normal_()* function in the library. During training, the network used the Adam optimizer (Kingma and Ba, 2014) with a learning rate of 0.0001 for the Davis and KIBA datasets and a learning rate of 0.001 for the other datasets to adjust the network parameters. To prevent overfitting, L2 regularization was added to the loss function. Each batch contained 256 samples, and the samples were randomly scrambled. Three hundred epochs were executed. Finally, the model was trained by minimizing the cross-entropy loss function.

$$L\left(y,\hat{y}\right)=-\left(ylog\hat{y}+\left(1-y\right)\log\left(1-\hat{y}\right)\right)+\lambda \sum {\left\|\Theta \right\|}^{2}$$

where y is the true value, $\hat{y}$ is the predicted value, λ is the regularization coefficient, and Θ is the network parameter. On the basis of the parameters in Table 1, we search the optimal value of λ by grid searching scheme within range of [-5,+5]. Empirical experiments show that setting the value of λ being -3 yield the best performances.

### Results

First, we conducted experiments on the BindingDB dataset extracted by Gao et al. According to the environment they set for the dataset, we utilized the same division to ensure that the data in the validation set did not appear in the training set, so that the experiment was closer to the real-world situation. During the training process, to prevent overfitting, we set the termination criterion according to the ACC evaluation index of the validation set. When the ACC of the validation set iterated for a certain number of steps and did not increase, the program terminated. To demonstrate the superiority of the model, we made a comparison with k-nearest neighbors (K-NN), a random forest (RF), L2, a support vector machine (SVM) and the BridgeDPI model (Wu et al., 2022a). The machine learning results for these methods were derived from the source paper on C.elegans and Human dataset (Tsubaki et al., 2019). We conducted experiments on BindingDB dataset. The table shows that on the BindingDB dataset, the AUC, Precision, Recall, and F1 of the proposed model reached 0.913, 0.888, 0.822, and 0.854, respectively. Our model outperforms traditional machine learning methods in AUC, Precision, and F1. The unsupervised K-NN method yielded lower results than the other models and methods, with AUCs and F1 scores of 0.858/0.814 and 0.860/0.858 on the C.elegans and human datasets, respectively. The effects of the RF, L2, and the SVM based on supervised learning were better. The AUC on the C.elegans dataset reached approximately 0.9, and the AUC on the human data exceeded 0.9. Compared with traditional machine learning methods, our model achieved the highest evaluation indicators on the C.elegans dataset, and its AUC, precision, recall, and F1 were 2.3%, 3.7%, 2.0%, and 2.9% higher than those of the second-best approaches, respectively. At the same time, our method performed slightly better on the human dataset. Since models such as K-NN, the RF, L2, and the SVM cannot obtain high-quality feature information, it is not easy for them to learn complex nonlinear DPIs. However, deep learning has strong feature extraction capabilities. Our model benefits from that. The Table 2 shows that our model doesn’t perform as well as BridgeDPI that extracts features from the biological perspective. Our model is similar to nature language processing in extracting featrues, and it’s indeed not as effective as BridgeDPI. However, our model has some advantages: when dealing with drug features and protein features, an attention mechanism is introduced to realize the key sites of drug-protein binding, thereby ignoring irrelevant site information and saving the screening time of drug-protein interactions. This has contributed to experts to identify drug-protein interactions.

**TABLE 2:** AUC, precision, recall, and F1 values obtained under different methods.

Models	AUC	Precision	Recall	F1
BindingDB dataset				
K-NN	0.776	0.762	0.791	0.776
RF	0.742	0.834	0.600	0.698
L2	0.737	0.784	0.646	0.709
SVM	0.805	0.770	0.858	0.811
BridgeDPI	**0.960**	0.883	**0.903**	**0.893**
Ours	0.913	**0.888**	0.822	0.854
C.elegans dataset				
K-NN	0.858	0.801	0.827	0.814
RF	0.902	0.821	0.844	0.832
L2	0.892	0.890	0.877	0.883
SVM	0.894	0.785	0.818	0.801
BridgeDPI	**0.996**	**0.980**	**0.970**	**0.975**
Ours	0.925	0.927	0.897	0.912
Human dataset				
K-NN	0.860	0.798	0.927	0.858
RF	0.940	0.861	0.897	0.879
L2	0.911	0.891	0.913	0.902
SVM	0.910	**0.966**	0.950	0.958
BridgeDPI	**0.990**	0.962	**0.965**	**0.963**
Ours	0.914	0.934	0.862	0.897

The best result was highlighted in bold, while the suboptimal is denoted by underline.

To further demonstrate the feature extraction advantages of deep models, we performed a multiclass prediction experiment on the Davis and KIBA datasets, and the results are shown in Table 3. The Davis and KIBA datasets possess continuous values, and the $pK_{d}$ values of Davis and the scores of KIBA are distributed as shown in Figure 4. We found their means μ and variances σ according to the relationship between the mean and variance. We divided the data into five categories: [μ−σ,μ+σ], [μ−2σ,μ+2σ], [μ−3σ,μ+3σ], [μ−4σ,μ+4σ], and other . According to our model, the classification effect of the test was relatively objective, and the ACC and AUC reached 0.850/0.705 and 0.841/0.813 on the Davis and KIBA datasets, respectively. AUC is the best on both Davis and KIBA datasets. ACC is lower on Davis dataset due to uneven data distribution and less data, which may affect our results. The results show that the proposed model is robust.

**TABLE 3:** Multi-classification results obtained on the Davis and KIBA datasets.

	ACC		AUC	
Models	Davis	KIBA	Davis	KIBA
K-NN	0.854	0.777	0.581	0.574
RF	**0.868**	0.811	0.610	0.591
L2	0.752	0.699	0.582	0.556
SVM	0.862	0.801	0.541	0.543
Ours	0.850	**0.841**	**0.705**	**0.813**

The best result was highlighted in bold, while the suboptimal is denoted by underline.

## Conclusion

In this work, we propose a model based on VAEs and attention mechanisms to predict DPIs. The high-level features of drugs and proteins are further extracted by a CNN and an attention mechanism. Experiments show that our method outperforms some base methods on the testing datasets and illustrates the powerful ability of deep learning to extract features. To further verify the robustness of the model, we perform a multiclass prediction experiment on the Davis and KIBA datasets. The final results of the experiment yield good metric values.

## Data Availability

Publicly available datasets were analyzed in this study. This data can be found here: https://www.bindingdb.org/bind/index.jsp, http://staff.cs.utu.fi/∼aatapa/data/DrugTarget/, https://wormbase.org//species/c_elegans#104--10.
